# Wavefront shaping improves the transparency of the scattering media: a review

**DOI:** 10.1117/1.JBO.29.S1.S11507

**Published:** 2023-12-09

**Authors:** Chunxu Ding, Rongjun Shao, Qiaozhi He, Lei S. Li, Jiamiao Yang

**Affiliations:** a Shanghai Jiao Tong University, School of Electronic Information and Electrical Engineering, Shanghai, China; b Shanghai Jiao Tong University, Institute of Marine Equipment, Shanghai, China; c Rice University, Department of Electrical and Computer Engineering, Houston, Texas, United States

**Keywords:** wavefront shaping, scattering media, time reversal, optical focusing and imaging

## Abstract

**Significance:**

Wavefront shaping (WFS) can compensate for distortions by optimizing the wavefront of the input light or reversing the transmission matrix of the media. It is a promising field of research. A thorough understanding of principles and developments of WFS is important for optical research.

**Aim:**

To provide insight into WFS for researchers who deal with scattering in biomedicine, imaging, and optical communication, our study summarizes the basic principles and methods of WFS and reviews recent progress.

**Approach:**

The basic principles, methods of WFS, and the latest applications of WFS in focusing, imaging, and multimode fiber (MMF) endoscopy are described. The practical challenges and prospects of future development are also discussed.

**Results:**

Data-driven learning-based methods are opening up new possibilities for WFS. High-resolution imaging through MMFs can support small-diameter endoscopy in the future.

**Conclusion:**

The rapid development of WFS over the past decade has shown that the best solution is not to avoid scattering but to find ways to correct it or even use it. WFS with faster speed, more optical modes, and more modulation degrees of freedom will continue to drive exciting developments in various fields.

## 1 Introduction

Light can carry and process information in parallel in multiple degrees of freedom, such as space, time, wavelength, amplitude, phase, and polarization. Therefore, it has been an essential tool for information observation, transmission, and compilation in the last several decades. Optical techniques are indispensable, from tracking the trajectory of stars in astronomy, to observing the microscopic structure of cells in biomedicine. But if light propagates in a scattering environment, such as disordered materials, biological tissues, and multimode fibers (MMFs), the inhomogeneous distributions of refractive indices will add random distortions to the carried information.^1^^–^^3^ This phenomenon significantly deteriorates the performance of traditional optical techniques. Biomedicine is particularly affected. High-resolution imaging and high-precision laser therapy often rely on ballistic photons or quasiballistic photons^4^^–^^6^ because they can achieve coherent and tight focusing. However, scattering in biological tissues causes the number of ballistic photons to decay exponentially with propagation depth, limiting optical focusing and direct imaging to a depth of ∼1 to 2 mm.^7^ At the depths of more than one optical transport mean-free path, ballistic photons are rare, and fully developed speckles become dominant. Thus for a long time, overcoming scattering to make the turbid medium “transparent” has been urgently needed.

The phenomenon of scattering events exhibiting determinism with the speckle decorrelation window is called the memory effect.^8^^–^^10^ Although the scattering transmission of ballistic photons is highly complex, there is no irreversible loss of the carried information. If the scrambled information in the scattered light can be successfully retrieved and rearranged, the “transparency” of the medium may be achieved. Based on this, Vellekoop and Mosk developed wavefront shaping (WFS).^11^ The phase control pattern of the spatial light modulator is optimized by an iterative algorithm, and the precompensation information is introduced into the input light.^12^^–^^15^ A focused spot can be successfully obtained behind the scattering medium. A transmission matrix (TM) model describing the linear relationship between the input and output light has also been established.^16^^–^^19^ By measuring the TM, the scattering distortion can be precompensated to obtain the desired output. In addition, time-reversing the scattered field can also restore the speckle to the original incident wavefront.^20^^–^^23^ In the past decade, thanks to the development of highly adjustable spatial light modulators, such as liquid crystal,^11^ microelectromechanical,^24^ and acousto-optic modulators,^25^ especially the increase in the number of controllable modes and modulation rates, a large amount of research on WFS has emerged.

WFS has provided a powerful tool for biomedicine. By manipulating large-scale optical modes with high resolution, WFS allows the construction of diffraction-limited spatiotemporal focusing behind opaque biological tissues.^26^ Furthermore, guidance with ultrasound, which produces a focus much deeper than light in the medium, can help to focus light inside turbid tissues without invasive manipulation.^27^ The high-resolution and high-contrast antiscattering focusing based on WFS increases the penetration depth of two-photon microscopy,^28^ optical coherence tomography,^29^ and fluorescence imaging,^30^ promising noninvasive diagnosis and treatment,^31^ and benefiting the development of phototherapy and optogenetics. MMF only has a diameter of a few hundred microns but can support tens of thousands of optical modes. But the inherent mode crosstalk makes it impossible to directly image but produces speckles similar to multiple scattering. WFS can compensate for the crosstalk, thereby subtly converting MMFs into endoscopic fibers for minimally invasive imaging of living cells and tissues. Thus WFS accelerates the deployment of MMFs to endoscopic imaging.^32^

In this review, we introduce the principles of WFS, including the concept of light scattering and three basic methods. Then we review the latest developments in the field of focusing, imaging, and MMF. Finally, we discuss the current challenges and prospects of WFS.

## 2 Basic Principles of WFS

### 2.1 Multiple Scattering

**Fig. 1:**
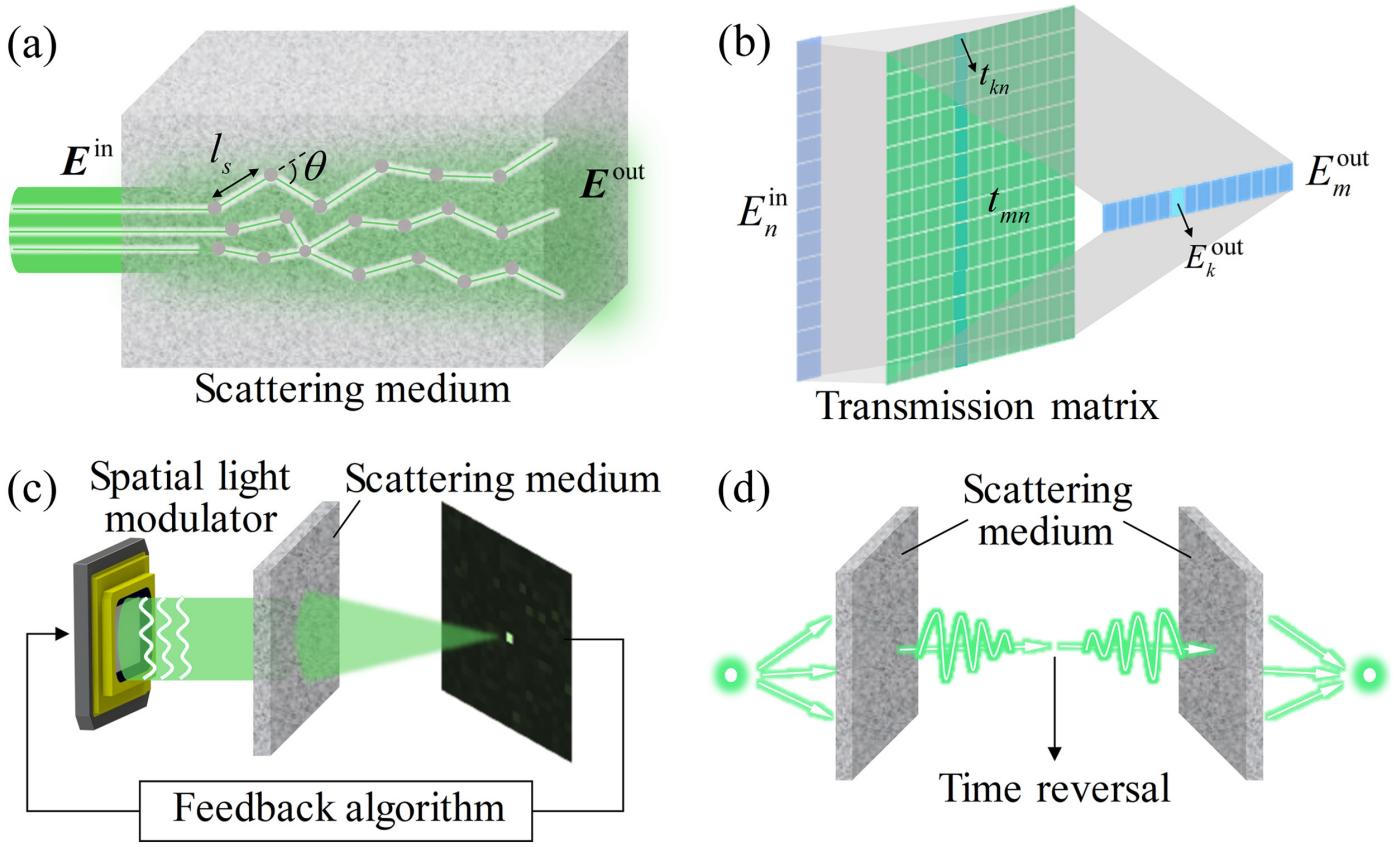
Principles of WFS: (a) propagation of light in a scattering medium. (b) Visualization of TM. The input and output optical fields are expanded as vectors. The field $E_{k}^{\text{out}}$ at the k’th output mode is linked to the input $E_{n}^{\text{in}}$ by a column $t_{mn}$ of TM. (c) The optimization method. The output field of the scattering medium is used as the feedback to optimize the modulation pattern of the spatial light modulator by the algorithm, and the desired output is finally obtained. (d) OPC. Time-reverse scattered field and recover the input field.

Affected by the inhomogeneity of the microscopic refractive indices of the medium, photons on different paths experience different phase delays, and the transmission directions are deflected to different degrees [Fig. 1(a)]. Therefore, ballistic photons decrease sharply during propagation, and the scattered light of different paths interferes randomly, forming a speckle pattern with chaotic intensity distribution.^1^ Generally, the absorption coefficient $\mu_{a}$ is used to describe a fractional decrease in the light intensity per unit distance traveled due to absorption, and the scattering mean free path of scattering $l_{s}$ is used to represent the average distance between two adjacent scattering events.^33^ $l_{s}$ quantifies the scattering strength of the scattering medium. After the distance known as the transport mean free path $l=l_{s}/(1-g)$, the propagation path of the scattered light becomes sufficiently random, completely losing correlation with the original trajectory.^2^ g is the anisotropy factor of the scattering medium and represents the average value of the cosine of the light deflection angle θ during the scattering events. The anisotropy factor of biological tissue is typically ∼0.9,^31^ which means that the forward scattering is dominant. The transport mean free path is typically 0.1 to 1 mm, and the observed speckle patterns are basically the result of multiple scattering.

Theoretically, the transport of light can be described by the wave equation, as can multiple scattering. However, the complexity of multiple scattering makes it nearly impossible to obtain a complete numerical solution. However, there are correlations between multiple scatterings. The same input can result in an unchanged speckle response. When the input changes slightly in tilt, the speckle pattern maintains a high degree of similarity, i.e., memory effect.^34^ It should be noted that the memory effect is valid within the speckle correlation time.^1^ Once there is a disturbance, the scattering transformation will quickly decorrelate. The transformation of the light field before and after scattering is deterministic. If the optimal inputs can be constructed according to the transformation, it will be possible to obtain the desired output through scattering.

Mesoscopic transport^35^ and random matrix theory^36^ provide a solution for the quantitative analysis of multiple scattering. In the spatial domain, the input and output fields of the scattering medium can be represented by $E_{n}^{\text{in}}$ and $E_{m}^{\text{out}}$, respectively (with n=1,…,N and m=1,…,M). n and m are the optical channels or modes. For a system with a surface area A, theoretically, the maximum number of spatial modes allowed for the input and output is $N_{m}\approx 2\pi A/\lambda^{2}$, where λ is the wavelength of light.^37^ In practice, N and M depend on the discrete resolution and the finite field. The transformation between input and output can be described by a linear complex TM T, i.e., $E_{m}^{\text{out}}=\sum t_{mn}E_{n}^{\text{in}}$ or $E^{\text{out}}={TE}^{\text{in}}$. The TM indicates that a channel of the scattered output is the weighted sum of all input channels.^16^ When the input is a plane wave, the output depends on the entire matrix. Since the results of multiple scattering are fully developed speckles, where channels or modes are highly uncorrelated, each element of the TM is close to a random variable, generally obeying a circularly symmetric Gaussian distribution. The TM expression can also be modified to represent a reflective matrix not limited to the transmissive forward scattering.^38^

### 2.2 WFS via TM Engineering

The TM describes the linear relationship between the light before and after scattering [Fig. 1(b)]. If each element of the matrix can be accurately obtained, all the scattering events can be analyzed, and any scattering output can be achieved using a specially designed input. Therefore, measuring the TM to compensate for the scattering distortion is the focus of WFS. At present, there are two major methods for measuring the TM of scattering media, namely the interferometric method^39^^–^^42^ and the noninterferometric method.^43^^–^^46^ The former is first demonstrated by Popoff et al.^16^ They loaded the orthonormal basis as the input to the spatial light modulator sequentially. The camera detected the interference between the speckle output of each basis and the additional reference beam. The complex amplitude information of the detection was analyzed by the phase-shifting method, and finally the TM was obtained by specific calculation.^16^ In Popoff’s method, since the reference beam and the signal beam came from the same path, it was also called self-reference measurement. Additionally, introducing an external reference for phase shifting or holographic measurement can also be used for the measurement. The noninterferometric method is first demonstrated by Drémeau et al., mainly relying on phase retrieval algorithms to infer the elements of the TM from a large number of input patterns and corresponding speckle outputs.^47^ The phase retrieval algorithms are mostly driven by Bayes’ theorem,^43^ and the measurement of TM is completed with many iterations. Due to the antinoise performance, the noninterferometric method is currently a popular research.

Once measured, the TM T can be used to analyze and control the light propagating through the scattering medium. By analyzing the eigenvectors of $T^{†}T$, the transmission eigenchannel, complete control of the light transmission in the scattering medium can be achieved.^48^ By inverting the TM via $E^{\text{in}}=T^{-1}E^{\text{out}}$, the input wavefront that should be modulated can be calculated according to the target output, or the hidden input can be recovered from the output speckle.^49^^,^^50^ In practice, the inverse of the matrix is often replaced by the conjugate transpose $T^{†}$ or some other operators.^51^ Although not exactly equivalent, it is more stable than direct inversion. Because the goal of antiscattering focusing is to form a single focus at one fixed target location, it is not necessary to measure the entire TM. The optimal input wavefront is only related to a certain column of the TM. Therefore, the measurement error tolerance is high. However, recovering the input image from the speckle, or constructing a specific output requires more accurate measurement, because almost all elements of the TM are used in the calculation. But due to various issues, such as the limited number of optical modes and unstable interferometry, the implementation of these complex tasks is quite difficult and often requires additional help from iterative or learning-based methods.^52^^,^^53^

In order to control more degrees of freedom of the scattered light, a lot of expansions of the complex amplitude TM method have been developed. For example, the control of polarization allows the construction of vector beams for optical tweezers,^54^ increasing the information capacity of MMFs.^55^ Based on the polarization coupling effect of scattering, and the fact that the scattered vector field is the summation of the intensities of two fully developed speckles in two orthogonal polarization states,^51^ researchers extend the linear transformation relationship to a two by two dimension,^56^ which means a pair of inputs in two orthogonal polarization states becomes a pair of scattered outputs. In addition to the spatial degrees of freedom, spectral and time resolved transmission matrices can also be obtained using monochromatic measurement at multiple frequencies,^57^^,^^58^ or low-coherence measurement in a broad spectrum of light.^59^^,^^60^ Then frequency-dependent focusing and spatiotemporal focusing can be achieved.

### 2.3 Iteration-Based WFS

Iteration-based WFS uses the field distribution at the target position as the feedback to search and optimize the modulation pattern of the spatial light modulator with iterative algorithms [Fig. 1(c)]. It can gradually optimize the incident light and guide the scattered light to generate the target field through the scattering medium.^11^^,^^61^ This method was the first demonstration of WFS. In the implementation of iteration-based WFS, taking focusing as an example, detection devices, such as cameras or photomultiplier tubes, are used to monitor and detect the light intensity of the target focusing position. The iterative algorithm takes the detected light intensity as feedback and completes the optimization.^61^^–^^65^ Finally, the one with the highest feedback is determined as the best modulation pattern, and a highly enhanced antiscattering focusing can be obtained.

The iteration-based method can be used to overcome the diffraction limit of a conventional imaging system and generate enhanced focusing,^66^^,^^67^ without complex interference techniques. For iteration-based WFS, imaging a large field-of-view often requires focus scanning.^1^^,^^68^ It generally relies on the memory effect. However, the effective range of memory effect is small, which leads to a limited imaging field of view and depth. Constructing a movable guide star, such as ultrasound,^69^ can achieve deep imaging without the aid of memory effects, but the focal spot is several orders of magnitude larger than the optical diffraction limited focus.^66^^,^^70^ The imaging resolution and contrast are typically insufficient. Recently, a model-based method has been proposed to achieve the focusing at any spatial location by calculating the refractive index model of the scattering medium.^71^ But this method requires strong prior knowledge, and its practicability needs further exploration.

The exploration of advanced noninvasive feedback is another important direction for iteration-based methods.^27^ In practice, cameras cannot be used to provide feedback inside the scattering medium. However, there is an urgent need to achieve optical focusing and imaging inside biological tissues. Therefore, internal guide stars have been proposed, such as fluorescence markers,^72^ photoswitchable proteins,^73^ magnetic particles,^74^ and microbubbles.^75^ The fluorescent marker was employed in the medium as the earliest guide star.^72^ The fluorescence signal outside the medium is detected as feedback to optimize the focused spot. In addition, people have proposed several noninvasive guide stars, such as coherence gating^76^ and particle displacement.^77^ Recently, acousto-optic feedback methods have been extensively studied,^66^^,^^69^^,^^78^^–^^80^ as ultrasound can propagate deep in biological tissue. In addition, the acoustic signal generated via the photoacoustic effect inside the scattering medium as the feedback was also explored.^29^^,^^81^ Moreover, people can also focus ultrasound in the medium to induce acousto-optic modulation to shift the light frequency and detect the shift as the feedback to achieve noninvasive focusing.^69^

### 2.4 Optical Phase Conjugation

The analytical solution of the wave equation has time-reversal symmetry.^37^ It means that if the phase conjugate field of the scattered field propagates back to the scattering medium, the initial incident field can be restored [Fig. 1(d)]. When the incident light comes from a specific point source, the time-reversed light is refocused at that location. This method based on time-reversal is called optical phase conjugation (OPC). OPC was first demonstrated in analog devices, such as acoustic time-reversal mirrors^82^^,^^83^ and optical nonlinear crystal.^84^^,^^85^ Digital methods of interference recording and spatial light modulation can be alternatives to analog methods to achieve optical focusing through turbid media.^20^

Compared with the TM and iteration-based WFS, the wavefront information to be modulated can be determined by OPC with only one measurement, significantly reducing the time consumption. It can achieve a fast response of millisecond or submillisecond level.^86^ But OPC also introduces a system alignment challenge. This challenge stems from two major implementation steps of OPC: recording of scattered optical fields, and playback of phase-conjugate optical fields. In a typical implementation, the camera records the interference patterns of the scattered light and the reference light, and the complex amplitude of the scattered light is calculated. Then the spatial light modulator displays the corresponding pattern to generate the phase-conjugate field and trace it back to the scattering medium. In this process, the pixels of the recording camera and the playback modulator need to be matched one by one, so that the time-reversal can be accurately achieved,^23^ adding complexity to the implementation of OPC. One possible solution is to design a unique device with both detection and modulation functions.^1^ In addition, to achieve the focus inside the scattering medium, the guide stars, such as ultrasound, are also required.^73^^,^^76^^,^^87^

## 3 Application of WFS

### 3.1 Focusing and Imaging

Clinical imaging technologies, such as ultrasound imaging^29^ and magnetic resonance imaging,^1^ can penetrate biological tissues to obtain macroscopic images. But their spatial resolution is orders of magnitude lower than optical microscopy. In general, the scattering properties in biological tissues are wavelength-dependent.^88^ As the wavelength increases, the transport mean free path becomes longer. Near-infrared light can reach a penetration depth of several millimeters to centimeters.^89^ But conventional optical techniques using visible or near-infrared light, such as multiphoton microscopy,^90^ still rely on ballistic photons, limiting the imaging depth to 1 to 2 mm. Using scattered light rather than the almost nonexistent ballistic light is a better choice to solve the problem in biological tissues. The rapidly developed WFS makes it possible to achieve focusing and imaging through thick scattering media and even in living tissues.

**Fig. 2:**
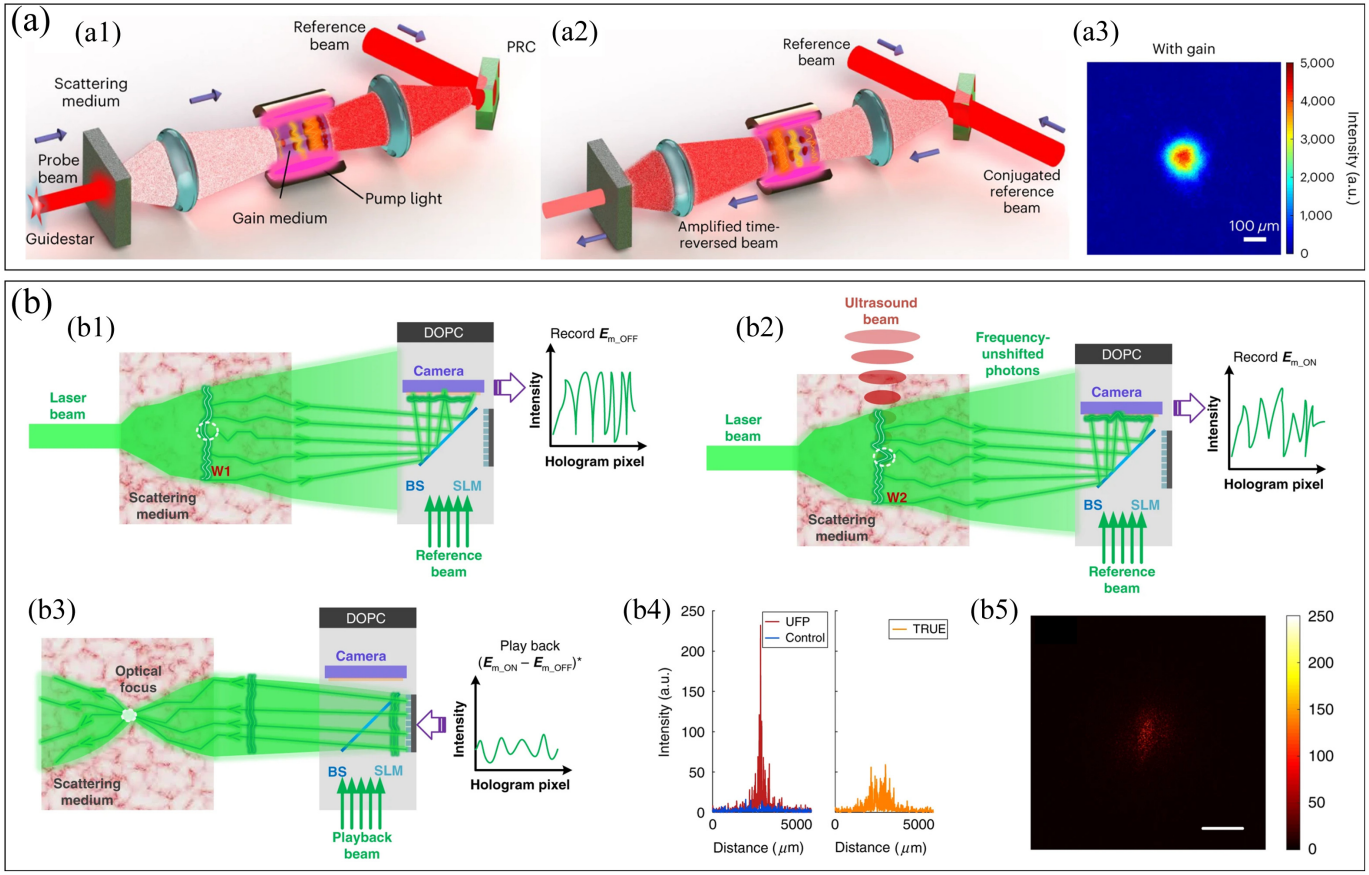
Fast and high-enhanced optical focusing. (a) Fast OPC: (a1) forward amplification and recording process based on stimulated emission light amplification; (a2) playback process based on time-reversal; and (a3) optical focusing results in a living-mouse ear. (b) Ultrasound-guided *in vivo* optical focusing: (b1) ultrasonic guide star switch off; (b2) ultrasonic guide star switch on; (b3) playback the differential field to achieve *in vivo* focusing; (b4) energy profile of focusing; and (b5) experimental focusing results. Panels (a1)–(a3) are from Ref. 96. Panels (b1)–(b5) are from Ref. 80.

Fast and highly enhanced focusing inside scattering media has been the focus of research. In general, the energy gain of light focusing is affected by the number of effective modulation modes. However, increasing the number of modes reduces the speed of focusing. This trade-off is particularly prominent in TM and iteration-based methods.^1^ Digital micromirror devices (DMDs) with high refresh rates are used to replace the traditional liquid crystal modulator, greatly improving the focusing speed.^18^ However, DMDs can only control the binary amplitude of light, then the focusing quality is reduced. A variety of binary coding methods have been proposed, such as Lee holographic method^91^ and superpixel method,^92^^,^^93^ to improve the modulation dimension of DMD to the complex amplitude. But these methods sacrifice the number of modes. In addition, polarization has also been taken into account, and WFS is carried out in two orthogonal polarization directions.^23^^,^^94^^,^^95^ On the one hand, the number of modes was cleverly increased by two times. On the other hand, it enables WFS to control polarization, another important degree of freedom of light. Recently, a WFS technique that simultaneously achieves high-speed, high-energy gain, and high-modulation mode number has been proposed^96^ [Fig. 2(a)]. By combining photorefractive crystal-based analog OPC and stimulated emission amplification, the focusing energy gain is increased by three orders of magnitude, and the speed is improved by more than 50 times. Light focusing in a living mouse ear has been achieved. It promises optical focusing for biomedical applications.

Focusing light in the scattering medium, such as biological tissue, requires guide stars *in vivo*.^27^ Since the scattering effect of ultrasound is about 1000 times weaker than that of light, a new concept of optical focusing based on ultrasonic encoding was first proposed and applied to WFS.^69^ It is noninvasive and moveable. Because this method uses the focus of ultrasound in the tissue to guide the focus of coherent light, the resolution of optical focus is limited by the ultrasonic focus.^42^ In order to improve the resolution, some methods, including variance coding time inversion^97^ and nonlinear ultrasonic guided iterative optimization^66^ have been proposed. These methods break the ultrasonic resolution limit and realize the optical diffraction-limited focusing. Since only a small amount of frequency-shifted first-order diffracted photons are useful for focusing, it reduces the focusing efficiency and peak-to-background ratio. Recently, it has been found that the brighter zeroth-order diffracted light, which was previously considered useless or even harmful, has a field perturbation effect^80^ [Fig. 2(b)]. When switching the ultrasonic guide star on and off alternately, the differential field can be detected, thereby guiding the light to focus to the location of the field disturbance, improving the efficiency and flexibility of light focusing.

**Fig. 3:**
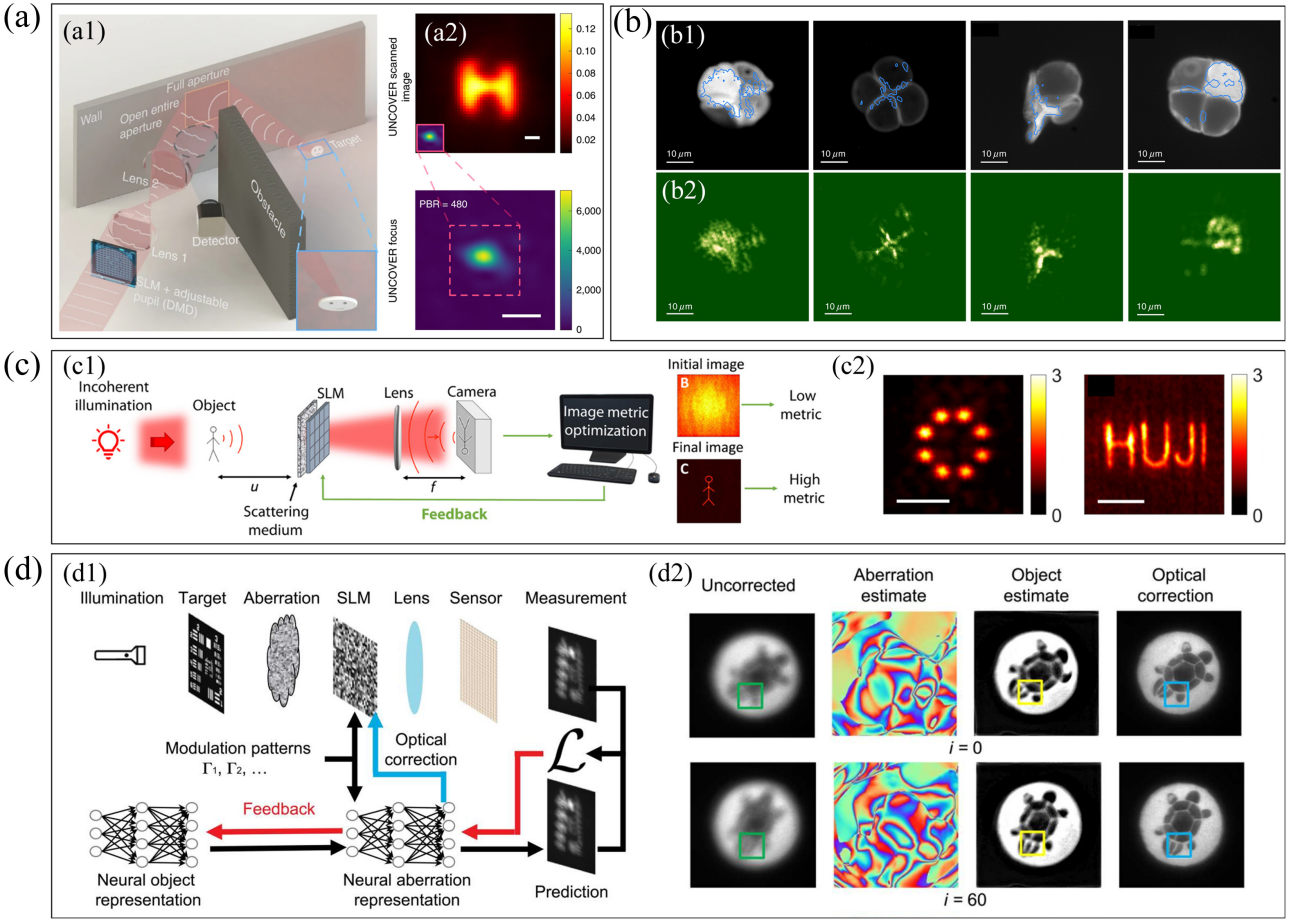
Optical imaging of WFS. (a) Non-line-of-sight imaging based on WFS: (a1) principle and (a2) imaging results and the scanning focus. (b) Fluorescence imaging based on memory effect: (b1) fluorescence images of different pollen grains without scattering and (b2) imaging results with scattering. (c) Image-guided WFS: (c1) principle and (c2) imaging results. (d) Learning-based WFS: (d1) principle and (d2) imaging results of dynamic objects under dynamic scattering aberrations. Panels (a1) and (a2) are from Ref. 99. Panels (b1) and (b2) are from Ref. 100. Panels (c1) and (c2) are from Ref. 101. Panels (d1) and (d2) are from Ref. 102.

The most straightforward method of translating focusing to imaging is raster scanning, which can be combined with fluorescent labeling to realize the imaging of microstructure by scanning the focus.^98^ It can also replace the time-gated method by modulating the light to compensate for the scattering of the wall and form a focus to scan hidden objects, to achieve non-line-of-sight imaging^99^ [Fig. 3(a)]. Because the focus of WFS can be close to the diffraction limit, the imaging resolution can be significantly improved. In addition, the memory effect makes it possible to achieve imaging without scanning focus^100^ [Fig. 3(b)]. This method can reconstruct the hidden objects in the scattering environment using the speckle correlation calculation method without complex optimization of the wavefront. However, it should be noted that such methods only can image small objects in the narrow-angle range where the memory effect takes effect.

In recent years, the development of deep learning has made a breakthrough in WFS.^103^^,^^104^ An image-guided WFS method was developed^101^ [Fig. 3(c)], which iteratively optimizes the phase of the spatial light modulator with the goal of maximizing the image quality metric (improved combination of entropy and variance). Diffractive-limited, high-contrast images of hidden objects from the very low-contrast speckle pattern can be recovered, even with the object outside the field of view of the memory effect. In addition, introducing neural networks to calculate complex optical aberrations from scattering media is also a promising direction^102^ [Fig. 3(d)]. Through backpropagation without guide stars, the antiscattering imaging of a wide field of view of dynamic scenes at diffraction-limited resolution can also be achieved.

### 3.2 WFS in MMF

Correcting aberrations inside MMF has also been studied via WFS. In general, an MMF has a diameter of only a few hundred microns but can support tens of thousands of optical modes. This advantage makes it an ideal device for endoscopic imaging. It also holds great potential to replace the coarse glass fiber bundles^105^ currently used for medical purposes. However, due to the mode coupling, meaning different modes occur in different phase delays, MMF shows the characteristics of the scattering medium. The light through MMF cannot be directly used to form an image but produces a disordered speckle. The conversion of MMF to endoscope imaging fiber using WFS has been widely studied.

**Fig. 4:**
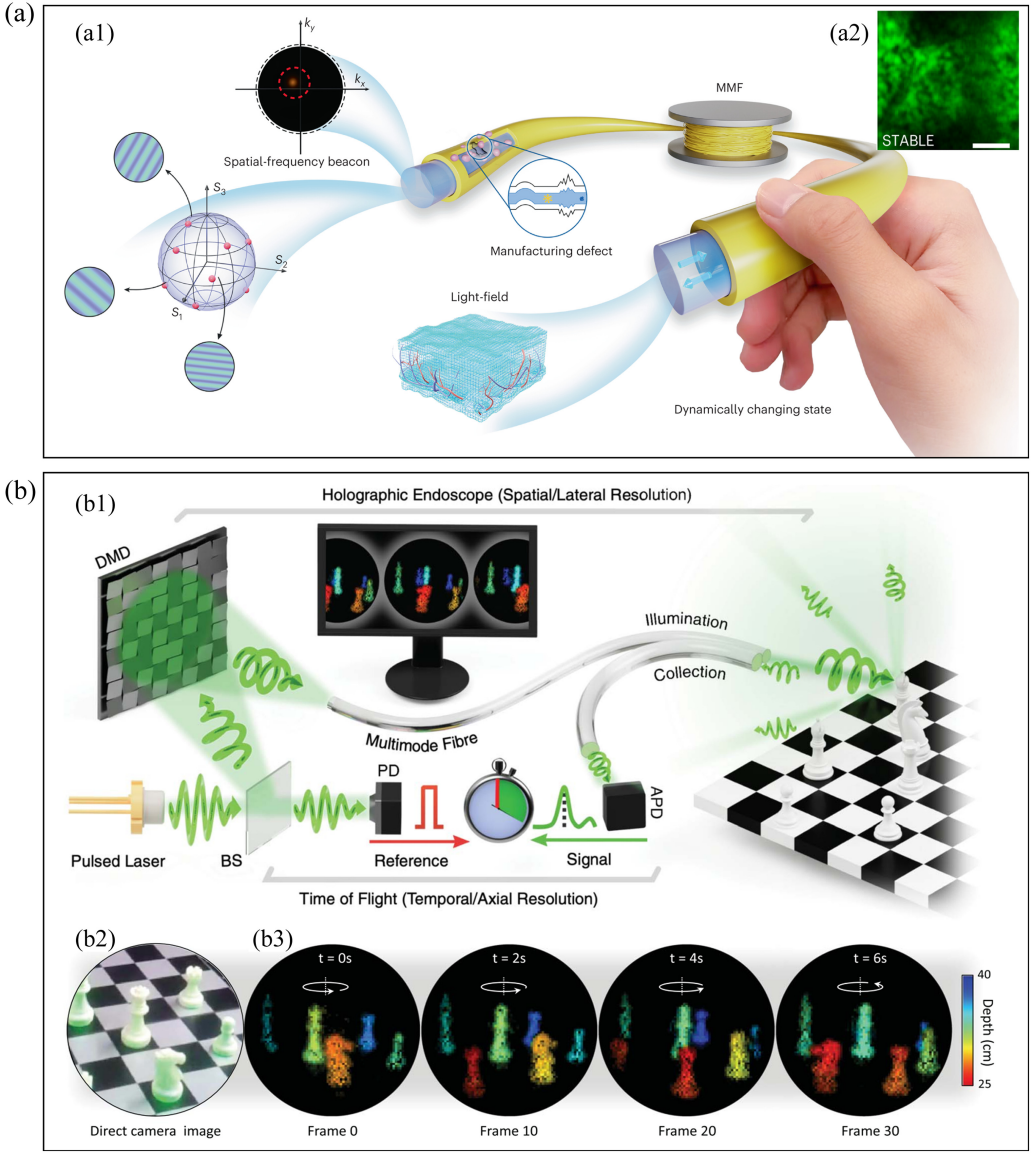
Imaging through an MMF. (a) Adaptive MMF endoscope: (a1) principle and (a2) endoscopic imaging results. (b) Three-dimensional far-field imaging through an MMF: (b1) principle; (b2) imaging target; and (b3) imaging results. Panels (a1) and (a2) are from Ref. 110. Panels (b1)–(b3) are from Ref. 111.

For the linear mode coupling process in MMF, the optical input and output can still be connected by the TM. Therefore, WFS is effective in MMF. In fact, methods, such as OPC,^28^ TM,^106^ and iterative optimization,^107^ have been applied in MMF-based endoscopy imaging, and *in vivo* imaging of animal neurons^108^ and blood cells^109^ has also been realized. However, these methods require MMF not to be deformed or bent, which limits applications in medical diagnosis. A spatial-frequency tracking adaptive beacon light-field-encoded endoscope has been proposed recently^110^ [Fig. 4(a)]. The special beacon intensity that reflects the correlation between the current bending states and the precalibrated TM is detected by the single-pixel detector. Adaptive tracking is completed by searching the TM that maximizes the intensity from the premeasured dataset. This method can improve the resolution of endoscopic imaging to subdiffraction-limited value with high stability and robustness against the movement and deformation of the fiber. In addition to endoscopic microstructure imaging, MMF has also been used in recent years for far-field imaging of three-dimensional (3D) real scenes^111^^,^^112^ [Fig. 4(b)]. Using WFS to correct the aberration of MMF, combined with the time-of-flight method, high-speed 3D imaging can be achieved, benefiting motion detection and tracking for industrial applications.

**Fig. 5:**
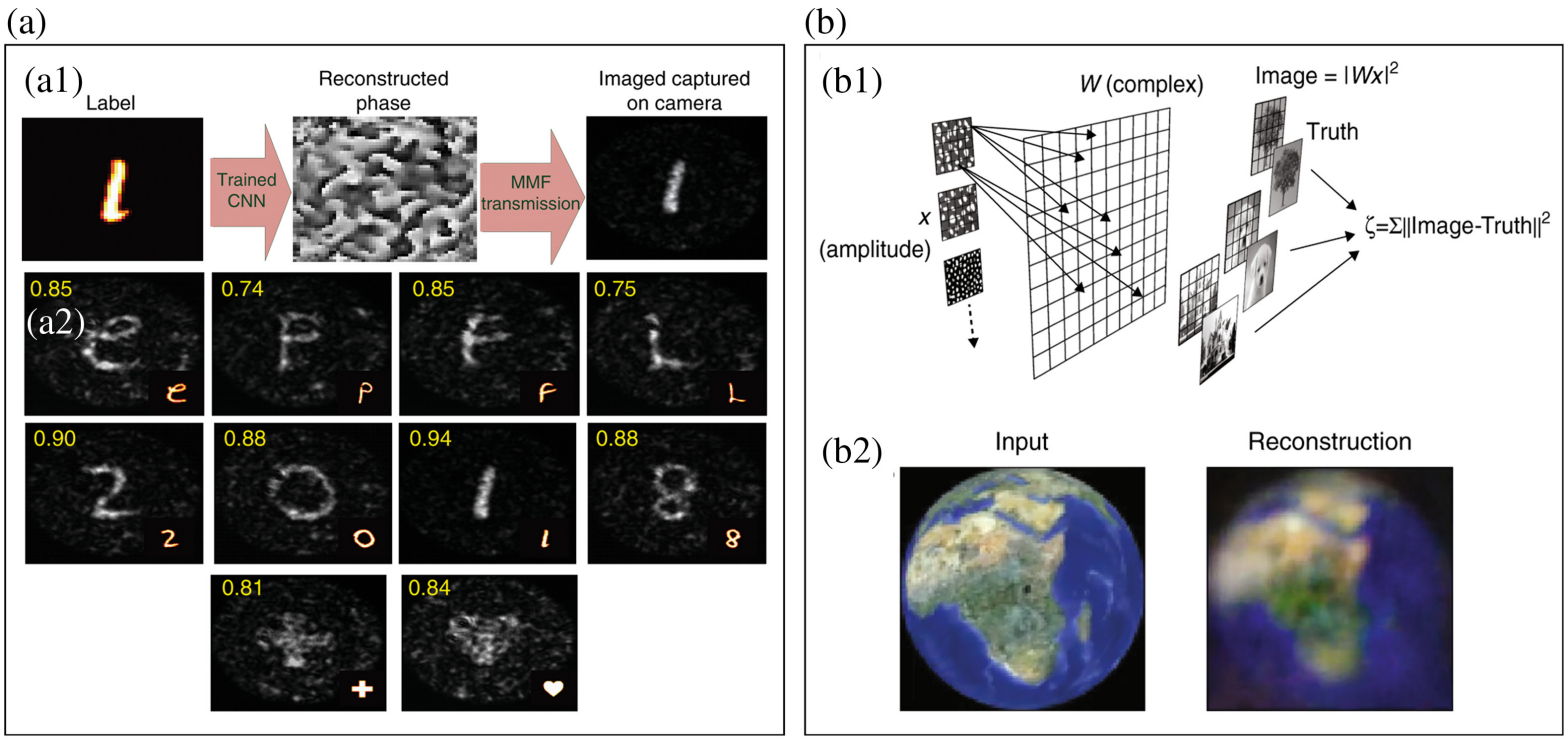
Smart imaging of MMF. (a) Learning-based imaging: (a1) flowchart and (a2) imaging results. (b) Statistics-based imaging: (b1) principle and (b2) input image and reconstruction results. Panels (a1) and (a2) are from Ref. 113. Panels (b1) and (b2) are from Ref. 114.

However, there are nonlinear effects^32^ between the speckle output and the input of MMF, which greatly reduce the effectiveness of the TM based on the linear premise. Deep neural networks have been shown to be useful for learning nonlinear processes in MMF.^104^ By training a conventional convolutional neural network,^113^ the input image can be recovered from the speckle without the unstable TM measurement [Fig. 5(a)]. However, the generalization of the neural network is limited, and it is usually only effective for the images highly similar to the training data set. Thus the performance is limited for the general purpose of image transmission. A method to reconstruct the complex inversion matrix of MMF by statistical means successfully realizes the transmission and imaging of full-color natural images with high frame rate and high resolution, without data-based learning^114^ [Fig. 5(b)]. Because this approach is based on a physically informed model of the imaging system that retrieves an approximation to TM, its generalization is better than that of the deep neural network approaches.

**Fig. 6:**
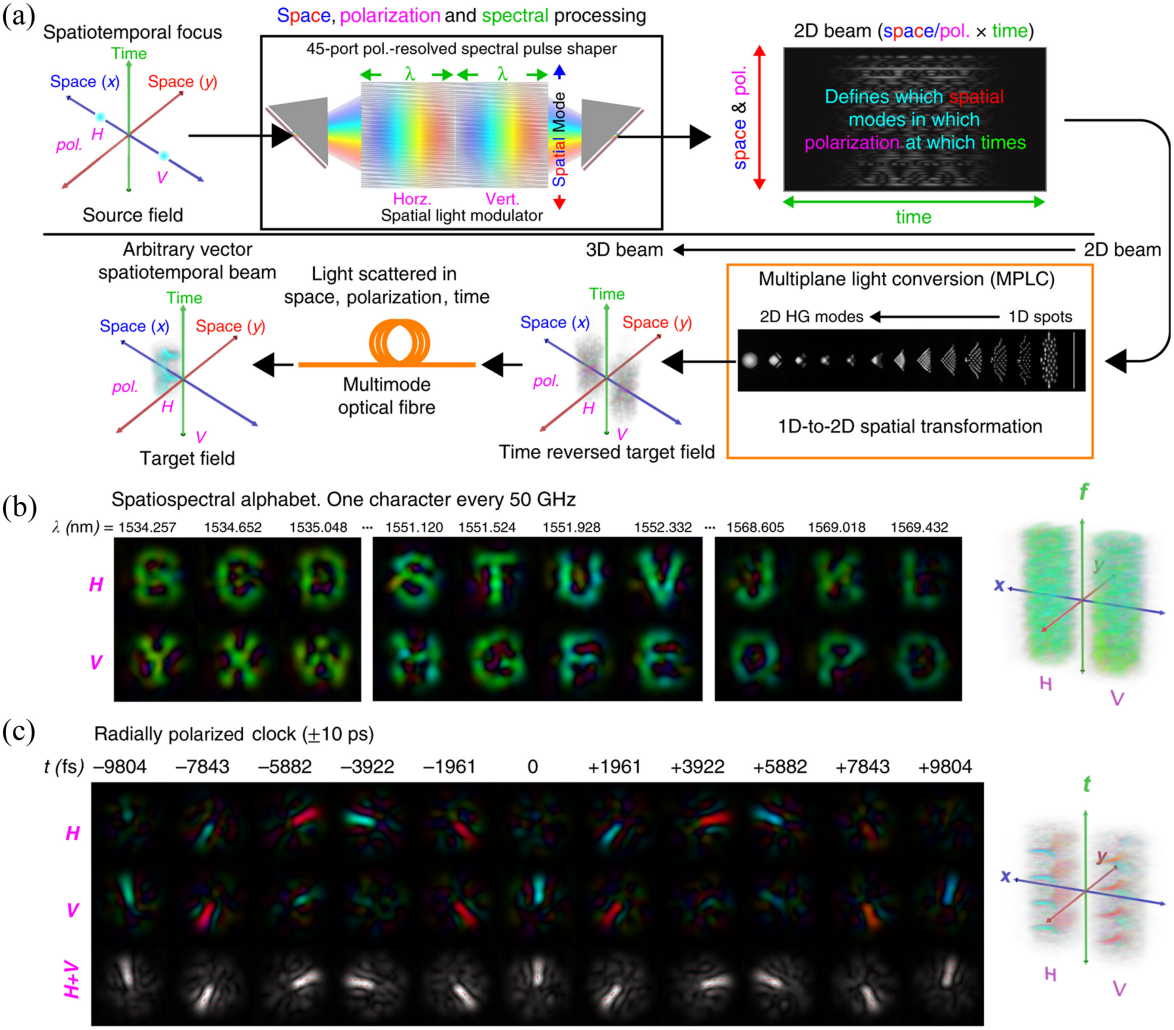
Multidegrees of freedom light control in MMF: (a) principle; (b) vector spectral light generation; and (c) optical field with space and polarization state changing with time. Panels (a)–(c) are from Ref. 117.

The mode coupling of MMF occurs in various degrees of freedom.^51^ In principle, to achieve full control of MMF, the modulated light needs to be completely reversed in time. This means that the modulated light is a volume field that should have arbitrarily specified amplitude, phase, and polarization at every point in space and time. However, this kind of full degree of freedom controlled field has been difficult to produce in research.^115^^,^^116^ Recently, a time-reversal device that can independently and simultaneously measure and control all classical degrees of freedom has been invented^117^ (Fig. 6). This method can generate a series of arbitrary two-dimensional vector fields at a high rate and is used for spatiotemporal control of light in MMF, including imaging, nonlinear optical phenomena, and micromanipulation. The modulation of all degrees of freedom could be an important direction of WFS development.

## 4 Discussion and Outlook

So far, WFS has shown unique advantages and potential in solving scattering problems. In this paper, the basic concepts and general methods of WFS, as well as the contributions and developments in focusing, imaging, and optical fiber endoscopy, are reviewed. It is worth mentioning that although dealing with scattering in biomedicine is the current focus, the applications of WFS are not limited to this.^118^^–^^121^ As mentioned in the introduction, in recent years, holographic display^122^^–^^124^ and optical communication^125^^–^^127^ have also been supported by the rapid development of WFS. However, at present, they are still in the experimental verification stage. For example, the holographic display using scattering can only generate a simple pattern composed of focused points, which is far from the commercial 3D digital holographic display.^128^

A key problem preventing WFS from practical applications is the trade-off between the speed and the number of modulation modes. The TM of the scattering medium changes with time, environmental disturbance, or bending deformation.^129^^,^^130^ In general, the decorrelation time of biological tissue is approximately milliseconds and decreases with the increase of tissue depth.^131^ In addition, for MMF, bending is basically unavoidable. Despite a lot of exploration, such as the development of modulators from liquid crystal modulators to DMDs with refresh rates of up to kilohertz,^24^^,^^132^ there is still a long way to go to achieve real-time correction of dynamic scattering. WFS that takes effect within the speckle correlation time will be the key to determining whether it can be applied.

In addition, the number of input and output optical modes of a scattering medium is huge, but the modes WFS can control are often limited or even insufficient. On the one hand, the number of input modes often depends on the number of effective modulation units, which is much smaller than the theoretical maximum number,^37^ which limits the performance of WFS. For example, the enhancement of antiscattering focusing is approximately linear with the number of modulation modes.^66^ On the other hand, the number of pixels of the camera is also limited, and the speckle imaging in the camera is a lowpass filtering process.^32^ Thus the measurement of all output modes is almost impossible. Currently, people often choose to trade the number of optical modes for the speed,^1^ because the increase of modes in most methods puts a significant burden on speed. WFS outside the speckle correction time window is no longer useful to correct the scattering.

Apart from the speed and mode numbers, the degree of freedom of control is also a key to WFS. Multiple scattering not only confuses light into speckles of randomly distributed intensity but also disturbs the information of almost all degrees of freedom of light.^51^ The ultimate ideal of WFS needs to control all the information of the light, including but not limited to spatiotemporal information in the scattering process, which will truly enable the “transparency” of the scattering medium. Currently, it is still a challenge to control the complex information of all the degrees of freedom independently for existing spatial light modulators. With the development of vector vortex beams,^54^ spectroscopy,^133^ and nonlinear optics,^134^ this challenge can potentially be overcome.

Data-driven learning-based methods bring new vitality to the development of WFS. Instead of relying on physical models, such as TM, a deep neural network model is used to learn compensating scattering aberrations from a large training data set and can achieve better performance. For example, it can learn how to correct dynamic scattering aberrations^102^ and the deformation of MMF.^114^ It can also enlarge the field of view limited by the memory effect, lower the requirement of scattering calibration, and improve the stability of the system.^135^ The relevant research is in the initial stage, and future engineering efforts are still needed.^1^ For example, training a neural network requires a large and diverse set of targeted data, resulting in expensive training and insufficient generalization. Learning-based imaging mostly stays in the two-dimensional plane, and how to translate it to depth-resolved volume imaging is still challenging. Meanwhile, since the training process requires direct access to the input and output planes, imaging objects inside the scattering medium presents another difficulty. Nevertheless, learning-based WFS is becoming the focus of research and is promising to bring about a new technological revolution in this field.

## 5 Conclusion

Scattering is a fundamental problem in the field of optics. Overcoming the limitation of scattering and achieving optical “transparency” in turbid media is always an urgent goal. The rapid development of WFS over the past decade has shown that the best solution is not to avoid scattering but to find ways to correct it or even use it. So far, there are many well-established methods to compensate scattering to construct a bright focus^136^^,^^137^ or to transmit images.^138^^,^^139^

In this paper, the basic principles, methods of WFS, and the latest applications of WFS in focusing, imaging, and MMF endoscopy are described. In addition, the present challenges and outlooks of WFS are discussed. Currently, there are three major aspects for the development of WFS: faster to adapt to dynamic scattering,^140^ more modes to meet the needs of actual scenarios,^96^ and more degrees of freedom to be controlled simultaneously.^95^ Fortunately, the introduction of data-driven learning-based methods may open up a new possibility for WFS.^141^ Although WFS is still not mature enough for practical applications, the development of this field is very encouraging. Various exciting results show the great potential that WFS will drive major developments in biomedicine, imaging, optical communication, and other fields.

## Data Availability

The code and data that support the findings of this study are available from the corresponding authors upon reasonable request.
