# Wavefront shaping improves the transparency of the scattering media: a review (Publisher’s Note)

**DOI:** 10.1117/1.JBO.29.S1.S19801

**Published:** 2024-02-05

**Authors:** Chunxu Ding, Rongjun Shao, Qiaozhi He, Lei S. Li, Jiamiao Yang

**Affiliations:** a Shanghai Jiao Tong University, School of Electronic Information and Electrical Engineering, Shanghai, China; b Shanghai Jiao Tong University, Institute of Marine Equipment, Shanghai, China; c Rice University, Department of Electrical and Computer Engineering, Houston, Texas, United States

## Abstract

The publisher’s note serves to clarify a correction to a figure in the published article.

This article [*J. Biomed. Opt.* **29**(S1), S11507 (2023) doi: 10.1117/1.JBO.29.S1.S11507 was originally published on 9 December 2023 with an error in Fig. 5. The caption for the figure was correct, but the image was mistakenly flipped, so that panels (a) and (b) were swapped. The figure was rectified and the article republished on 15 December 2023.

